# Effects of Robot-Assisted Gait Training with Body Weight Support on Gait and Balance in Stroke Patients

**DOI:** 10.3390/ijerph19105814

**Published:** 2022-05-10

**Authors:** Wonho Choi

**Affiliations:** Department of Physical Therapy, Gachon University, Incheon 21936, Korea; whchoi@gachon.ac.kr; Tel.: +82-32-820-4423; Fax: +82-32-820-4420

**Keywords:** stroke, body weight support, robot-assisted gait training

## Abstract

This study investigated the effects of robot-assisted gait training with body weight support on gait and balance in stroke patients. The study participants comprised 24 patients diagnosed with stroke. Patients were randomly assigned to four groups of six: robot A, B, C, and non-robot. The body weight support (BWS) for the harness of the robot was set to 30% of the patient’s body weight in robot group A, 50% in robot group B, and 70% in robot group C. All experimental groups received robot-assisted gait training and general physical therapy. The non-robot group underwent gait training using a p-bar, a treadmill, and general physical therapy. The intervention was performed for 30 min a day, five times a week, for 6 weeks. All participants received the intervention after the pre-test. A post-test was performed after all of the interventions were completed. Gait was measured using a 10 m Walking test (10MWT) and the timed up and go (TUG) test. Balance was assessed using the Berg Balance Scale (BBS). Robot groups A, B, and C showed significantly better 10MWT results than did the non-robot group (*p* < 0.5). TUG was significantly shorter in robot groups A, B, and C than in the non-robot group (*p* < 0.5). The BBS scores for robot group A improved significantly more than did those for robot groups B and C and the non-robot group (*p* < 0.5), indicating that robot-assisted gait training with body weight support effectively improved the gait of stroke patients.

## 1. Introduction

According to the Global Burden of Disease, the number of deaths due to stroke per 100,000 people is 42.56 [1]. Moreover, 3.3 million die due to ischemic stroke annually, ranking second among causes of death [1]. One in four people above 25 years of age worldwide will experience a stroke in their lifetime, and the stroke incidence is increasing annually [1,2], resulting in a global burden of more than USD 891 billion [1].

Stroke is defined as a clinical manifestation of a sudden onset of local or global impairment of brain function due to vascular causes that can last more than 24 h or even result in death [3]. Neurological and functional defects persist after experiencing strokes [2]. Daily living ability is reduced owing to balance and gait problems that result from muscle weakness, motor control, pain, stiffness, and poor balance ability [2,4], while the risk of falls is increased by the impediment of mobility recovery.

Gait disturbance is the biggest obstacle limiting the daily activities of stroke patients [5]. Abnormal gait patterns appear after stroke onset, and walking distance is also limited [6]. Most patients display slow walking speeds and decreased endurance [6]. The decrease and loss of walking ability cause long-term disability in stroke patients, making treatment difficult [7] and, thus, making walking one of the most important treatment goals in the rehabilitation of stroke patients. Gait recovery after neurological damage is the primary goal of rehabilitation, and significant time and effort are required to improve gait ability.

Neurodevelopment treatment (NDT) is a well-known treatment for the recovery of patients with neurological deficits and gait disorders after stroke, the goal of which is to convert abnormal movements into normal movements [8,9,10].

Body-weight-supported treadmill training has traditionally been used for gait rehabilitation [11,12]. In this training, one or more therapists partially assist the patient in supporting their weight and then guide the patient’s movements and gait on a treadmill [13]. Body weight-supported treadmill training offers numerous advantages in patient gait rehabilitation because gait motion becomes more accurate and repetitive than before body weight support [14]. However, body-weight-supported treadmill training entails a great deal of physical labor and a high risk of injury because the therapist alone must support the patient’s weight [15].

Robot-assisted gait training supplements the limitations of NDT and body-weight-supported treadmill training [10,16]. In robot-assisted training, the patient’s body weight is supported by a harness system, eliminating the need for therapist assistance, as in body-weight-supported treadmill training [16]. Robot-assisted gait training is characterized by repeatability and accuracy [17]. The repeatability is achieved by thoroughly controlling the range of motion and stride length of the hip, knee, and ankle joints through robot hardware [17,18]. Once the robot hardware is set, treatment continues in the same training environment with repeatability unless the therapist intervenes as part of the treatment [19]. Accuracy is achieved through the robotic exoskeleton, as it is set on the patient according to the size suitable for the patient’s individual physical characteristics [19]. Furthermore, the therapist can provide precise stimulation by controlling the hardware of the robot. Based on such repeatability and accuracy, the robot-assisted gait-training device affects the coordination between the lower extremities in the gait of hemiplegic patients, thereby affecting postural control and adaptation and improving the patient’s gait [20,21]. Furthermore, the robot hardware allows control of the treadmill, harness system, and robot frame so that various techniques can be applied to the patient based on their needs [19]. Such a device can prevent fatigue and injuries in therapists and provide a wide range of gait-training environments for patients [13,20]. Furthermore, the robot offers effective treatment by providing precise, repetitive motions and a joint range of motion by controlling the robot’s hardware [17,22]. However, clear treatment guidelines have not yet been devised for robot-assisted gait training. There are no specific guidelines for the adjustable variables within the robot’s software, such as what speed is appropriate for the patient, how much of the patient’s weight should be supported for an optimal therapeutic effect, or what joint angle has the best effect on the patient’s gait. Despite its benefits, robot-assisted gait training has not been well established, either technically or conceptually, and must be investigated in future research. Therefore, this study aimed to investigate whether robot-assisted gait training improves gait and balance in stroke patients, as well as to examine the difference in treatment effects according to the extent of weight support within each robot treatment group.

## 2. Materials and Methods

### 2.1. Ethical Approval

The study was conducted in accordance with the guidelines of the Declaration of Helsinki and was approved by the Gachon University Institutional Review Board (Seongnam, Korea) (1044396-202112-HR-239-01). The purposes of the study and protection of the privacy of participants were fully explained. Once the participants agreed to participate in the study, they signed an informed consent form before the beginning of the study.

### 2.2. Participants

Twenty-seven hospitalized chronic stroke patients were willing to participate in the study, and three patients who did not meet the inclusion criteria were excluded. Thus, 24 patients were randomly selected based on the selection criteria. The selection criteria of the study participants were as follows: patients diagnosed with a stroke more than 6 months after onset; patients with no difficulty in following the therapist’s instructions with a score of ≥ 24 on the mini-mental state examination (MMSE); patients without orthopedic problems, heart disease, and circulatory problems; and patients able to walk >10 m using orthosis or mobility aids. Patients with open skin disease, severe fixation stiffness, height < 125 cm, weight > 135 kg, or Modified Ashworth scale (MAS) of G1+ or higher on the affected side were excluded.

### 2.3. Procedure

Twenty-four stroke patients were randomly assigned into four groups—robot A (*n* = 6), robot B (*n* = 6), robot C (*n* = 6), and non-robot (*n* = 6) groups—using simple randomization methods.

The general anthropometric characteristics were measured before the start of the experiment. Baseline measurements for the 10 m walking test (10MWT), timed up and go (TUG) test, and Berg Balance Scale (BBS) were measured. The rehabilitation duration for both groups was a total of 120 min rehabilitation a day, five times a week for 6 weeks. Both groups underwent their respective interventions for 60 min each in the morning and 60 min in the afternoon to minimize physical fatigue. They underwent intervention for 60 min regular physical therapy in the morning with an experienced physical therapist. Then, all robot groups underwent robot-assisted gait training with 30% (group A), 50% (group B), and 70% (group C) weight-bearing for 30 min. A 30 min session of robot-assisted gait training did not include the time needed to put on and take off the Lokomat. The non-robot group received gait training using a treadmill for 30 min. Additionally, both groups received standard one-on-one rehabilitation with an experienced physical therapist who specialized in training NDT for 30 min. Then, 10 MWT, TUG, and BBS were measured after 6 weeks of intervention in all groups.

### 2.4. Outcome Measures

#### 2.4.1. 10 m Walking Test

The 10MWT was used to assess gait speed [23]. The participant was asked to walk a 14 m walkway, including an acceleration for the first 2 m and a deceleration for the last 2 m, as fast as possible [24]. For evaluation, only the time taken to walk the middle 10 m was measured using a stopwatch [24]. The test–retest reliability was good (ICC = 0.87–0.88) and the intra-rater reliability was excellent (ICC = 0.95–0.99) in chronic stroke patients [25].

#### 2.4.2. TUG Test

The TUG test was used to assess basic mobility and balance [26]. The participant was asked to stand up from an armchair, walk 3 m directly, return, walk to the chair again, and sit down [27]. “Normal mobility” indicated that the time taken was < 10 s; “Good mobility” indicated that the time taken was < 20 s; “Limited mobility” indicated that the time taken was < 30 s; “Dependent mobility” indicated that the time taken was > 30 s [28]. The TUG test has excellent test–retest reliability (ICC = 0.95) in chronic stroke patients [26].

#### 2.4.3. BBS

BBS is used to assess balance and risk of falls [29] and contains 14 items, each scored from 0 to 4 [29]. A total score of “41 to 56” indicates good balance, “21 to 40” indicates acceptable balance, and “0 to 20” indicates balance impairment [30]. The test–retest reliability of BBS in chronic stroke patients was excellent (ICC = 0.98) [30]. The Korean version of BBS has excellent inter-rater reliability (ICC = 0.97) and intra-rater reliability (ICC = 0.95~0.97) [31].

### 2.5. Intervention

#### 2.5.1. Robot-Assisted Gait Training

**Figure 1:**
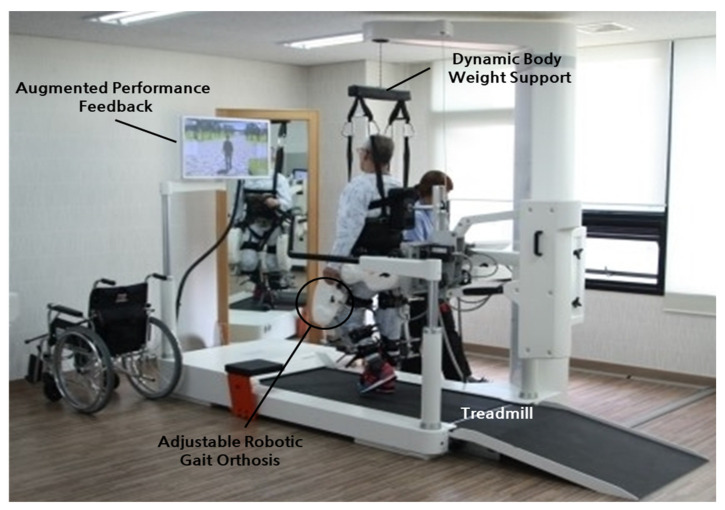
Robot-assisted gait training.

The Lokomat^®^ PRO (Hocoma AG, Zurich, Switzerland) was used for robot-assisted gait training. Lokomat^®^ PRO consists of a harness system that supports the patient’s weight, a robot frame structure, a treadmill system that guides the correct movement and alignment of the lower extremities during walking, an augmented reality (AR) program screen linked with the robot (simulation screen), and a robot hardware control system that controls the harness system, robot frame structure, and treadmill system (Figure 1).

Robot-assisted gait training was conducted as follows. First, the patient’s weight, height, and leg length were measured using a tape measure. The robotic exoskeleton was set up for the patient after the measured values were entered into robot hardware. The belt was then placed on the patient by using a suspension device, and the harness lifted the patient. While the patient was suspended approximately 10 cm above the floor, the robotic exoskeleton was placed on the patient in the order of hip, knee, and ankle joints. The patient’s joint position was aligned according to the movement of the robot, and the treadmill was operated. After adjusting the speed of the treadmill and the speed of the robot’s movements, the suspended patient was slowly lowered to adjust the height at which they were able to walk on the treadmill. Robot-assisted gait training was performed after setting the maximum speed according to the patient’s performance level determined by a Hocoma-certified trainer using a Hocoma manual. The treadmill started at a comfortable speed before accelerating to the maximum speed previously set.

Robot-assisted gait training was conducted for 30 min, and the weight-bearing capacity of the patient supported by the harness was set to 30% of the body weight in robot group A, 50% in robot group B, and 70% in robot group C. The degree of body weight support was chosen based on the most frequently used degree in clinical research studies on weight-supported gait training after stroke [32].

During robot-assisted gait training, the therapist set the gait speed within a range that did not affect patient performance. AR was enabled in robot-assisted gait training through an avatar for gait motion in conjunction with a monitor by using a built-in sensor, and the same level of game/exercises AR performance was applied equally to all robot-assisted gait training groups.

#### 2.5.2. Regular Gait Training

The non-robot group received regular gait training on the treadmill, an exercise load device that ran on a rotating crawler belt and was used to improve the gait of patients. The treadmill speed was set to the point at which the patient’s stride length and the treadmill belt speed matched the intervention of the therapist. Moreover, the patient’s gait status was continuously monitored while the therapist remained with the patient for 30 min to ensure safety.

#### 2.5.3. Neuro-Development Treatment

NDT focused on the treatments of secondary disorders that occur in patients due to damage to the central nervous system, decreased muscle strength, decreased range of motion, limited functional movement, decreased balance, and gait disturbance. The NDT technique used in the present study was performed based on Gjelsvik [32]. Treatment was tailored to each patient to improve gait and balance. The treatment involved the use of both the affected and unaffected sides. Efforts of pelvic tilt facilitation, trunk control, and weight transfer strategies were performed in supine, sitting, and standing positions. Treatment was conducted after improving walking ability by aligning the patient’s body, and retraining was conducted for muscles that are not used in walking to improve strength and engagement in incorrect movements. All groups received NDT from an experienced NDT-qualified therapist.

### 2.6. Statistical Analysis

Statistical analysis was performed using SPSS software (version 25.0; IBM, Armonk, NY, USA), and data were summarized as means and standard deviations (SDs). The normal distribution of variables was examined using the Shapiro–Wilk test, and all outcome variables were normally distributed. A chi-squared test and one-way analysis of variance (ANOVA) were performed to compare the general characteristics of the participants among the groups. One-way ANOVA was conducted to determine whether there were any differences in changes in outcome variables between pre- and post-intervention among the groups. Post hoc comparisons using the Tukey HSD test were performed when significant group main effects were detected. A paired *t*-test was conducted to determine any changes in outcome variables between pre- and post-intervention in each group. The level of significance was set at α = 0.05.

## 3. Results

Twenty-four stroke patients completed the study. The baseline characteristics were comparable among the four groups (Table 1). No serious adverse events related to the intervention were observed. The general characteristics of the participants are presented in Table 1.

Table 2 shows the outcome variables before and after the intervention in the four groups. There was a significant improvement in 10 MWT after the 6-week intervention in all robot groups (*p* < 0.05) but no significant difference was observed in the non-robot group (*p* > 0.05). Moreover, there was a significant difference in the changes in 10 MWT among the groups (F_(3,20)_ = 21.93, *p* < 0.001). The robot A group showed the greatest improvement, followed by robot B and C groups (*p* < 0.05).

Regarding the TUG test, a significant improvement was observed after the 6-week intervention in robot A and B groups (*p* < 0.05), while no significant improvement was found in robot C and non-robot groups (*p* > 0.05). There was a significant difference in the changes in TUG test scores among the groups (F_(3,20)_ = 30.62, *p* < 0.00). Post hoc analysis revealed that the robot A group showed a significant change in the TUG test score after 6 weeks of intervention compared to those of all other groups (*p* < 0.05).

Similarly, a significant improvement in BBS was found after the 6-week intervention in the robot A group (*p* < 0.05), while no significant improvement was found in the robots B, C, and non-robot groups (*p* > 0.05). The TUG test scores were significantly different among the groups (F_(3,20)_ = 17.32, *p* < 0.00). Post hoc analysis showed that the robot A group showed a significant difference in changes in BBS score after the 6-week intervention compared to all other groups (*p* < 0.05).

## 4. Discussion

The present study investigated the effect of training using a robot-assisted gait-training device on the gait and balance in stroke patients and the influence of the degree of weight-bearing capacity on the treatment effect of the robot-assisted gait-training device. As per our results, the groups that received robot-assisted gait training showed significant improvements in the 10MWT (straight walking ability) and TUG tests (functional walking ability) compared to the non-robot group, regardless of the weight-bearing capacity. Furthermore, robot group A, for which the weight-bearing capacity was set to 30%, displayed a significant improvement in balance.

The 10MWT and TUG tests were used to evaluate gait ability, and the robot group showed significant improvements in 10MWT compared to the non-robot group. These results are similar to those of the study by Peurala (2005), who reported a 24% improvement in walking ability following the application of robot-assisted gait training compared to the control group [33]. Furthermore, in Schwarz’s (2009) study of acute stroke patients, the robot-assisted gait training group significantly improved compared with the general gait training group, supporting the results of this study [34].

The TUG test scores significantly increased in robot groups A and B. However, a previous study that compared a robot-assisted gait training group with visual biofeedback and a control group that only received assisted gait training reported no significant difference in TUG test scores between the groups (Ham, 2015) [35]. Schwarz’s (2009) study reported no significant differences in the TUG test when comparing a weight-supported treadmill training group with a general gait training group [34]. Robot-assisted gait training with 30% (group A) and 50% (group B) weight bearing revealed significant improvements in the TUG test in the current study, which may be due to less weight bearing, as excessive weight support reduces ground reaction force (GRF) and sensory feedback [36]. This result is supported by the results of a study on hemiplegic stroke patients which reported improved functional gait ability in the group with a change in weight-bearing capacity (Barbeau, 2003) [37]. Moreover, the TUG test, unlike 10MWT, does not simply test the straight gait, which requires patients to walk straight to the destination and back, but rather evaluates functional movement performance, including curves and complex motions such as standing up and sitting down [38].

Regarding balance function, there was a significant improvement in BBS in the 30% body weight-bearing group (robot group A). Dias’s (2007) study reported a significant increase in BBS in both robot-assisted gait training and general gait training, but the high therapeutic effect was maintained for > 3 months only for robot-assisted gait training [39]. This may be because the stabilization of the patient’s trunk is provided by the robotic exoskeleton, and a uniform gait-training environment is provided through the controlled robot hardware, thereby promoting physical muscle activity through trunk stabilizer muscles [40]. Although the study by Ham (2015) reported that the improvement in balance with robot-assisted gait training with visual feedback was not significant, this report differs from this study, as it did not consider body weight support as a variable [35].

The reason why the training results varied depending on the amount of body weight support was as follows. Humans are subject to the gravitational force of the Earth, and as much as a human pushes down on the Earth with their weight, the Earth pushes back with afferent information referred to as GRF from the ground [41]. Humans receive this afferent information and control their bodies accordingly [42]. In case of excessive body weight support, such as 70% or 50% of the body weight, the afferent information received from the Earth is reduced [36]. Excessive changes in afferent information, such as excessive body weight support, cause instability and difficulty in promoting sensory information [42]. In other words, it is difficult to develop the ability to set the direction of the body and secure stability so that the balance control mechanism can be properly used in an environment in which gravity acts. Therefore, robot-assisted gait training with appropriate weight bearing can minimize instability by securing postural alignment and trunk stability. It can also contribute to improvement in gait by providing stability to certain body parts and the mobility necessary for functional activities of other body parts [17,40]. It should be noted that, in this study, within the training period, patients continued training at the initially set weight-bearing capacity, and there was no change in weight-bearing capacity until the end of the study period. However, in the study by Peurala (2005), when patients’ weight-bearing capacity was reduced from the initial 30% to 10% during the study, balance ability significantly improved in robot-assisted gait training [33]. Thus, a comparative study between a group with a change in weight-bearing capacity during the study period and a group for whom it is not changed should be conducted in the future.

This study had some limitations that need to be addressed. Although the balance and mobility functions were assessed, detailed physical function test such as the Fugl–Meyer assessment was not performed. Moreover, excessive weight support can cause difficulty in promoting information, but the present study did not measure the degree of sensory impairment directly. Further studies should measure these variables to differentiate more accurate patient characteristics to investigate the effect of robot-assisted training with different degrees of body weight support. In addition, the fact that robot-assisted gait training groups may have experienced a psychological placebo effect compared with the non-robot group, which may have affected the results, cannot be excluded. Lastly, the study has a relatively small sample size of 24 patients. Thus, the results are difficult to generalize and interpret. Further studies should investigate the different types of robot-assisted gait training with a sufficiently large sample by improving upon these limitations.

## 5. Conclusions

The results of this study indicate that robot-assisted gait training with body weight support is helpful in improving the straight-line walking ability of patients with chronic stroke. Furthermore, robot-assisted gait training with 30% weight bearing can improve functional gait and balance ability. Appropriate weight bearing for a positive effect on improving gait and balance in stroke patients should be considered.

## Figures and Tables

**Table 1:** General characteristics of participants (*N* = 24).

	Robot A (*n* = 6)	Robot B (*n* = 6)	Robot C (*n* = 6)	Non-Robot (*n* = 6)	*p*
Age (years)	52.7 ± 15.4	54.7 ± 12.3	59.5 ± 15.3	61.4 ± 9.7	0.463
Sex, *Females*, *n* (%) *	2 (33.3)	4 (66.7)	3 (50.0)	3 (50.0)	0.760
Height (cm)	163.2 3 ± 7.6	168.8 ± 9.4	163.7 ± 3.8	165.7 ± 7.9	0.555
Weight (kg)	57.5 ± 11.7	65.8 ± 4.1	61.7 ± 6.7	68.8 ± 10.3	0.153
Affected side, *left*, *n* (%) *	3 (50.0)	3 (50.0)	3 (50.0)	3 (50.0)	0.999
Onset (months)	20.2 ± 10.5	16.3 ± 9.5	16.8 ± 8.6	16.8 ± 6.9	0.834
K-MMSE (scores)	25.8 ± 1.2	26.5 ± 1.4	25.3 ± 1.2	25.8 ± 0.8	0.397

Abbreviations: K-MMSE: Korean-mini mental state examination; * except where indicated otherwise, values are presented as the mean ± SD.

**Table 2:** Outcome variables before and after the interventions among the groups (*N* = 24).

		Robot A (*n* = 6)	Robot B (*n* = 6)	Robot C (*n* = 6)	Non-Robot (*n* = 6)	F(*p*)
10MWT	Pre-test	24.7 ± 4.7	33.8 ± 6.6	32.5 ±7.1	22.3 ± 3.8	21.93 (0.000) (A > B > C > N)
	Post-test	15.5 ± 3.6	27.1 ± 7.6	28.6 ± 8.5	21.6 ± 4.4	
	△ pre-post	9.2 ± 1.9 **	6.7 ± 2.0 **	3.9 ± 1.9 *	0.7 ± 1.9	
TUG	Pre-test	25.4 ± 3.6	33.4 ± 7.4	35.6 ± 7.8	25.8 ± 4.9	30.62 (0.000) (A > B, C, N)
	Post-test	17.4 ± 2.9	31.4 ± 8.5	34.8 ± 8.8	24.8 ± 4.8	
	△ pre-post	8.0 ± 1.8 **	1.9 ± 1.8 *	0.8 ±1.6	0.4 ± 1.0	
BBS	Pre-test	33.3 ± 3.1	36.7 ± 3.3	34.0 ± 4.3	34.3 ± 2.7	17.32 (0.000) (A > B, C, N)
	Post-test	37.2 ± 2.8	36.8 ± 3.8	33.7 ± 4.2	34.5 ± 3.3	
	△ pre-post	3.8 ± 1.3 **	0.2 ± 1.2	0.3 ± 1.2	0.2 ± 0.8	

Abbreviations: 10MWT: 10 m walking test; TUG: Timed up and go; BBS: Berg Balance Scale; A: Robot A, B: Robot B, C: Robot C, N: Non-Robot, * significant difference between pre- and post-intervention at the 0.05 level; ** significant difference between pre and post at the 0.001 level. △ changes.

## Data Availability

The datasets generated during the current study are available from the corresponding author upon reasonable request.
